# Outcome of Endometrial Cancer Stage IIIA with Adnexa or Serosal Involvement Only

**DOI:** 10.1155/2011/962518

**Published:** 2011-05-04

**Authors:** Jan J. Jobsen, Lambert Naudin ten Cate, Marnix L. M. Lybeert, Astrid Scholten, Elzbieta M. van der Steen-Banasik, Job van der Palen, Marika C. Stenfert Kroese, Annerie Slot, Eltjo M. J. Schutter, Sabine Siesling

**Affiliations:** ^1^Department of Radiation Oncology, Medisch Spectrum Twente, Haaksbergerstraat 55, 7513 ER Enschede, The Netherlands; ^2^Laboratorium Pathologie Oost Nederland, 7512 ER Enschede, The Netherlands; ^3^Department of Radiation Oncology, Catharina Hospital, Eindhoven, The Netherlands; ^4^Department of Clinical Oncology, Leiden University Medical Center, 2333 ZA Leiden, The Netherlands; ^5^Arnhem Radiotherapeutic Institute, Arnhem, The Netherlands; ^6^Department of Research Methodology, Measurement, and Data Analysis, Faculty of Behavioral Science, University of Twente, 7500 AE Enschede, The Netherlands; ^7^Department of Clinical Epidemiology, Medisch Spectrum Twente, 7513 ER Enschede, The Netherlands; ^8^Radiotherapy Institute Steden en Omstreken, Deventer, The Netherlands; ^9^Radiotherapy Institute Friesland, Leeuwarden, The Netherlands; ^10^Department of Obstetrics and Gynaecology, Medisch Spectrum Twente, 7513 ER Enschede, The Netherlands; ^11^Comprehensive Cancer Centre North East, Enschede/Groningen, The Netherlands

## Abstract

*Objective*. The aim of this study is to look at possible differences in outcome between serosa and adnexal involvement stage IIIA endometrial carcinoma. *Methods*. 67 patients with stage IIIA endometrial carcinoma were included, 46 with adnexal involvement and 21 with serosa. A central histopathological review was performed. *Results*. The 7-year locoregional failure rate was (LRFR) 2.2% for adnexal involvement and 16.0% for involvement of the serosa (*P* = .0522). The 7-year distant metastasis-free survival was 72.7% for adnexal involvement and 58.7% for serosa (*P* = .3994). The 7-year disease-specific survival (DSS) was 71.8% for patients with adnexal involvement and 75.4% for patients with serosa. *Conclusion*. Endometrial carcinoma stage IIIA with involvement of the adnexa or serosa showed to have a comparable disease-specific survival. Locoregional control was worse for serosa involvement compared to adnexa.

## 1. Introduction

The majority of patients with endometrial cancer are diagnosed without evidence of extra uterine spread, leading to only 10–15% of the patients with stage III disease [1]. In 1988, the International Federation of Gynecology and Obstetrics (FIGO) mandated the staging of endometrial cancer be changed from a clinical staging system to a surgical staging system because of the inaccuracies of the former. Staging of the disease is since then based on pathological criteria after initial resection and evaluation.

According to FIGO 1997 stage IIIA endometrial carcinoma is defined as tumor involvement either as direct extension or metastasis to serosa, parametria, adnexa, and/or cancer cells in peritoneal washings [2]. Due to this broad definition, patients with stage IIIA form a heterogeneous group. Different types of tumor involvement within this heterogeneous group might have impact on prognostic factors and outcome. Due to the heterogeneity of the tumor characteristics in this stage and the low incidence only small series have been published. FIGO has recently updated the staging system with a significant change to the staging of endometrial carcinoma stage IIIA. The presence of abnormal cells in peritoneal washings no longer affects staging. This resulted in a less heterogeneous stage IIIA.

We had the opportunity to do a multi-institutional study on stage IIIA with isolated involvement of the serosa and adnexa. The aim of this study is to reveal possible differences in outcome between isolated serosa and adnexal involvement and determine possible prognostic factors in two homogeneous groups of patients with stage IIIA endometrial carcinoma based solely on involvement of the adnexa or the serosa.

## 2. Patients and Methods

All patients with FIGO stage IIIA, according to the 1997 and 2008 FIGO staging, endometrial carcinoma endometrioid type, based on isolated adnexa or serosal involvement were identified in six radiotherapy departments in the Netherlands over the period 1987 through 2005. All institutes had the opportunity to send in patients covering the above-mentioned period, which resulted in a total of 93 patients. 

The clinical data of the patients were collected from the charts: age at diagnosis, surgical procedure, histology, grade, depth of myometrium invasion, lymph vascular space involvement (LVSI), cervical involvement, positive peritoneal washing, adjuvant radiotherapy features, follow-up data (date and site of recurrence), and vital status.

A central histopathology review of all 93 patients was performed at the Laboratory of Pathology Oost Nederland by a single pathologist. All pathologic sections were reviewed on histology, grade, depth of myometrium invasion, LVSI, extent of cervical involvement, and extension of the tumor outside the uterus. 

The presence of a positive peritoneal washing was not routinely looked at and primarily not used as a variable for staging, but as an independent variable in the analysis.

Patients included in this study had only isolated involvement of the adnexa or the serosa. Patients with multiple involved sites were excluded. Involvement of both serosa and adnexa was also an exclusion criterion, leaving 67 patients for analysis after pathological review.

### 2.1. Treatment

All patients were primarily operated on. Standard surgery for clinical stage I endometrial carcinoma was total abdominal hysterectomy and bilateral salpingo-ooforectomie (TAH + BSO). In case of a suspected clinical stage II, the surgery was TAH + BSO and staging lymphadenectomy or Wertheim ****radical hysterectomy. Sixty-three patients (94%) had TAH + BSO, two patient underwent TAH with staging procedure, one patient had a vaginal hysterectomy, and one patient had a supracervical hysterectomy. 

Preoperative imaging to identify occult metastatic disease was not standard. Pathological stage IIIA endometrial carcinoma after TAH + BSO was a generally accepted indication in The Netherlands for postoperative external radiotherapy with or without vaginal vault irradiation, depending on the extension of the tumor and/or the radiotherapy department policy.

Nearly all patients, 95.5% (64/67), received postoperative pelvic external radiotherapy. The target volume included the upper two third of the vagina and the regional nodes. The upper border was defined at the L5-S1 interspace; the caudal border was defined to be the inferior margin of the obturator foramen. The lateral borders included the widest opening of the bony pelvis with a 1.5 cm margin. The external dose ranged from 30.0 to 46.0 Gy in 1.8–2.3 Gy fractions 4-5 times a week. Of the 64 patients, the majority (82.8%) received 46.0 Gy in 2.0 Gy fractions 5 times a week. From the 64 patients with postoperative pelvic radiotherapy, 25.0% (16/64) received a boost to the vaginal vault by brachytherapy and 7.8% (5/64) by external radiotherapy. The brachytherapy was fourteen times an application by vaginal cylinder and two times by ovoid's. The external boost was twice 20.0 Gy and three times 14.0 Gy, all in 2.0 Gy fractions.

Three patients from one center received total abdominal external radiotherapy of 20.0 Gy in 1.0 Gy fractions followed by a boost to the pelvic with doses of 20.0–24.0 Gy in 2.0 Gy fractions. Two of the three also received a boost by brachytherapy through vaginal cylinder. 

No adjuvant systemic therapy was given.

### 2.2. Statistical Methods

Time to recurrence and last date of followup were calculated from the time of surgery. To test for between-group differences for categorical data, Chi-square tests were used. The locoregional failure rate (LRFR) is defined as the number of vaginal and/or pelvic recurrences. Distant metastases were regarded as all extra pelvic recurrences, for example, abdomen, para-aortal, liver, lung, and bone. The distant metastasis-free survival (DMFS) was defined as survival without distant metastasis. The endpoint for the survival analysis was disease-specific survival (DSS), with censoring at date of the last contact or death due to other causes than endometrial carcinoma. Survival statistics were calculated by the method of Kaplan and Meier. For comparison of survival distributions the log-rank test was used. In univariate analysis we used the following variables: age, type of surgery, grade of differentiation, degree of cervical involvement, presence of positive peritoneal washing, presence of lymph vascular space involvement (LVSI), degree of myometrium involvement, type of adjuvant radiotherapy, use of adjuvant brachytherapy, and total external radiation dose. Variables that were univariately related to the outcomes of interest (*P* < .05) were entered in a multivariate Cox regression analysis.

The primary analyses were conducted with all 67 patients comparing adnexa and serosal involvement. A secondary analysis was performed with adnexa and serosal involvement separately. 

All analyses are based on the histopathologically reviewed data.

All analyses were performed using STATA [3]. 

## 3. Results

The tumor and treatment characteristics of all 67 patients, 46 with involvement of adnexa and 21 with serosa, are shown in Table 1. Age ranged from 40 to 87 years for all 67 patients with a median of 67 years. The median age for patients with involvement of the adnexa was significantly younger than the age of patients with involvement of the serosa (63.5 versus 71-years, resp.). The followup ranged from 3 to 217 months with a median of 56 months.

### 3.1. Locoregional Recurrence

The incidence during followup of locoregional recurrence was 2.2% (1/46) for patients with adnexal involvement and 14.3% (3/21) for patients with serosal involvement, which was not significantly different (*P* = .13). The 2-, 5-, and 7-year LRFR was 2.2% for adnexal involvement, and 10.0%, 16.0%, and 16.0% for involvement of the serosa, respectively. The log-rank test for equality of survival functions was significant for involvement of the cervix (*P* = .0098) and borderline significant (*P* = .0522) comparing adnexa and serosal involvement.

In a separate univariate analysis for patients with involvement of the serosa, only involvement of the cervix (*P* = .004) was significantly related to locoregional failure. None of the patients without cervical involvement showed locoregional failure. One patient with endocervical glands involvement and two patients with stroma involvement showed locoregional failure.

### 3.2. Distant Metastasis

**Figure 1:**
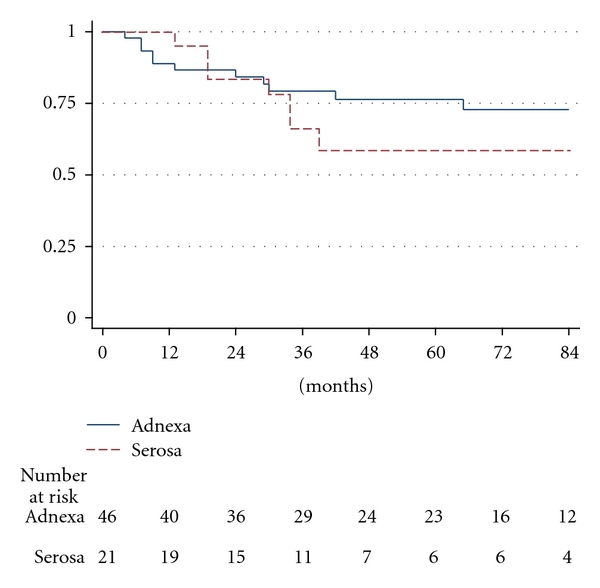
Distant metastasis-free survival for endometrial carcinoma stage IIIA according to involvement of the adnexa or serosa (log rank *P* = .3994).

The incidence of distant metastasis was 26.1% (12/46) for patients with adnexal involvement and 33.3% (7/21) for patients with involvement of the serosa (*P* = .54). The 2-, 5-, and 7-year DMFS was 84.3%, 76.4%, and 72.7% for adnexal involvement and 83.6%, 58.7%, and 58.7% for serosal involvement, respectively (Figure 1). 

In univariate analysis for all patients presence of LVSI (*P* < .001) and myometrium infiltration of >50% (*P* = .011) were significantly related to DMFS. Involvement of adnexa versus serosa was not significant (*P* = .40). In a multivariate analysis only the presence of LVSI (HR 3.1; 95% CI 1.03–9.11; *P* = .043) was significant. 

Separate analysis for patients with adnexal involvement showed grade of differentiation (*P* = .019), LVSI (*P* < .001), degree of myometrium infiltration (*P* = .012), and type of postoperative radiotherapy (*P* = .041) to be significantly related to DMFS. Due to small numbers, multivariate analysis was not reliable.

**Figure 2:**
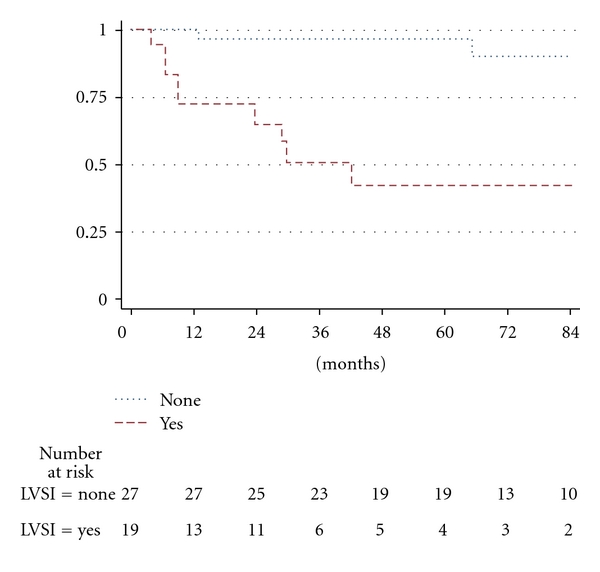
Distant metastasis-free survival for endometrial carcinoma stage IIIA with involvement of the adnexa according to presence of lymph vascular space involvement (log rank *P* = .0002).

The 7-year DMFS for the presence of LVSI versus none with involvement of the adnexa was 42.6% versus 90.6% (Figure 2). 

Analysis for patients with involvement of the serosa showed that none of the variables were significantly related to DMFS.

### 3.3. Disease-Free Survival

The 2-, 5-, and 7-year disease-free survival (DFS) was 84.3%, 76.4%, and 76.4% for patients with adnexal involvement and 84.3%, 59.6%, and 59.6% for patients with serosal involvement. Of the patients with adnexal involvement 12 showed distant metastasis, and one of those also showed a pelvic recurrence. Of those with serosal involvement 7 showed distant metastasis, and of those 3 patients also showed a vaginal recurrence.

In univariate analysis for all patients only LVSI (*P* < .001) and myometrium involvement (*P* = .015) were significantly related to DFS. Grade of differentiation showed borderline significance (*P* = .056). Involvement of adnexa or serosa did not show significant relationship with DFS. In multivariate analysis only presence of LVSI (HR 3.6; 95% CI 1.07–12.0; *P* = .038) was significant.

From the 46 patients with involvement of the adnexa 11 (23.9%) developed recurrences, of which one patient showed both locoregional and distant metastasis. Separate univariate analyses in patients with involvement of adnexa showed grade of differentiation (*P* = .009), LVSI (*P* < .001), myometrium infiltration (*P* = .019), and type of postoperative radiotherapy (*P* = .026) to be significantly related to DFS. Due to small numbers, multivariate analysis was not reliable.

Of the 21 patients with involvement of the serosa seven patients (33.3%) showed recurrences, of which three showed both locoregional and distant metastasis. In a univariate analysis only cervical involvement was significantly related to DFS (*P* = .012).

### 3.4. Disease-Specific Survival

**Figure 3:**
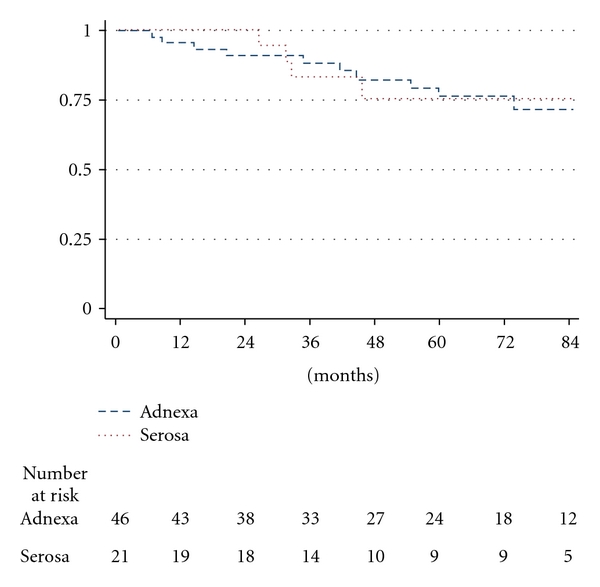
Disease-specific survival for endometrial carcinoma stage IIIA according to involvement of the adnexa or serosa (log rank *P* = .9194).

The 2-, 5-, and 7-year DSS was 91%, 76.3%, and 71.8% for patients with adnexal involvement and 100%, 75.4%, and 75.4% for patients with serosal involvement (Figure 3).

In a univariate analysis for all patients grade of differentiation (*P* = .0091), peritoneal washings (*P* = .038), and myometrium infiltration (*P* = .043) were significantly related to DSS. In a multivariate analysis including the above-mentioned variables only grade showed significance. Grade 3 had higher risk of death due to the cancer compared to grade 1 (HR 9.5; 95% CI 1.70–53.35; *P* = .010) and grade 2 compared to grade 1 (HR 6.2; 95% CI 1.18–33.14; *P* = .031).

Separate univariate analyses for patients with involvement of the adnexa showed grade of differentiation (*P* = .004), peritoneal washings (*P* = .024), LVSI (*P* = 0.001), myometrium infiltration (*P* = .021), and type of postoperative radiotherapy (*P* = .047) to be significant. Due to small numbers, multivariate analysis was not reliable.

In a univariate analysis for patients with involvement of the serosa none of the variables showed significance.

## 4. Discussion

Our study showed that patients with stage IIIA endometrial carcinoma with involvement of adnexa or the serosa had comparable disease-specific survival of 71.8% and 75.4% at 7 years, respectively. DMFS and DFS showed that patients with involvement of the serosa had a slightly worse outcome, but this difference was not significant. Locoregional control was excellent for patients with involvement of the adnexa, being only in 2.2% of the patient's recurrences, but was worse for patients with serosal involvement (14.3% recurrences).

The management and outcome for patients with stage IIIA endometrial cancer in the literature are diverse and unclear, due to the heterogeneity of the tumor characteristics in this stage and the low incidence leading to overall small series. Since several reports have shown that positive peritoneal cytology is not an independent prognostic factor if endometrial cancer is limited to the uterus, this item in this study is not regarded as a stage IIIA factor [4, 5]. This is in accordance with the new FIGO staging. Positive peritoneal washing is used as a variable comparable to myometrium involvement in the analysis.

In 2006 Randall published the results of a phase III trial comparing whole-abdominal irradiation versus chemotherapy for stage III and IV endometrial carcinoma [6]. They looked at stage III, incorporating IIIA, B, and C, and also used whole-abdominal irradiation. The heterogeneity of stage III and the different irradiation make it difficult to compare our results with this trial. 

 In our multi-institutional study we were able to comprise a relative large homogeneous group of patients with isolated involvement of the adnexa and also a group with only involvement of the serosa. To overcome the disadvantage of multi-institutional, a central pathological review by one pathologist was performed. Also the primary treatment being surgery and adjuvant radiotherapy was fairly homogeneous.

Most studies looking at prognostic factors for patients with stage IIIA endometrial carcinoma with involvement of the adnexa and/or serosa include also patients with other extra uterine spread, which makes it hard to compare our results with the literature [7–13]. 

Only few have looked at possible predictive or prognostic factors in these subgroups of stage IIIA [10]. This is of course due to the low incidence. As we know from the FIGO classification and have seen in our previous study, stage IIIA endometrial carcinoma is a heterogeneous group of patients [14]. We showed in our previous study that the number of involved sites is a strong negative prognostic factor. The majority show involvement of the adnexa alone or in association with other sites outside the uterus.

Several studies showed patients with extra uterine disease limited to the adnexa to have 5-year DFS ranging from 71 to 86% [12, 13, 15–18]. Preyer et al. in a retrospective study of 36 patients with adnexal involvement only and 10 patients with serosal involvement only found a median survival for both of 56.7 and 115.8 months, respectively [8].

Connell et al. found a 70.9% 5-year DFS for patients with solitary adnexal involvement with twenty patients [10]. He concluded that the relatively poor outcome seen in these patients is the result of known risk factors including differentiation grade, LVSI, and presence of additional sites of extra uterine disease. 

The DFS and DSS rates found in our study were comparable to rates reported previously. 

In contrast to most studies we had a relatively large group of patients with only adnexa or serosal involvement, making it possible to look at possible prognostic factors. In our separate analysis of 46 patients with involvement of the adnexa only, we were able to show in multivariate analysis LVSI and myometrium infiltration of the tumor to be independent prognostic factors, although we have to be aware of the small numbers resulting in large confidence intervals. Recurrences outside the pelvic area were the main problem for those patients. We had only one locoregional recurrence compared to 12 outside the pelvic area. Whether this excellent locoregional control is due to the treatment or surgery plus radiotherapy or inherent to the tumor characteristics cannot be answered in this study. On the other hand looking at the rate of recurrences outside the pelvis, adjuvant systemic therapy should be considered for those patients. These results also question the necessity of adjuvant radiotherapy for patients with solitary adnexal involvement and no presence of LVSI or myometrium infiltration <50%. Although the numbers are small, the differences in DMFS and DSS are highly significant. Randomized studies regarding treatment for these patients are not possible taking into account the low incidence. Further retrospective or prospective studies might answer this question.

Mariani et al. in a retrospective study of 14 patients with adnexa or serosal involvement or both observed a 57% 5-year DFS [7]. From those 14 patients 6 had serosal involvement. The 5-year DFS with serosal involvement compared to none was 17% and 87.5%, respectively. In a retrospective analysis of 19 patients with endometrial carcinoma stage IIIA Ashman et al. looked at outcome and prognostic factors [13]. Fifteen of those had solitary serosal involvement and showed a 41.5% 5-year DFS. Seven of those developed distant recurrences, and only one pelvic recurrence was seen. In our analysis of 21 patients with involvement of the serosa only we had a 59.6% DFS, but we were not able to distillate any prognostic factor in multivariate and univariate analysis. Recurrences outside the pelvic area (33.3%) were the main problem compared to 14.3% pelvic recurrences.

Patients with adnexa or serosal involvement have been shown to benefit from radiation therapy for locoregional control [19]. For patients with isolated serosal involvement; there is a decreased rate of pelvic recurrences with radiotherapy [13].

In this study recurrences were mainly outside the pelvis. Eighteen (26.9%) patients showed recurrences outside the pelvis, and from those 18 patients four also had a locoregional recurrence. From the point of locoregional control one can argue that postoperative radiotherapy to the pelvis results in a good locoregional control, which is consistent with others. Only this does not prove that adjuvant radiotherapy should be the treatment of choice. Looking at the results of this study and if we are able to minimize the side effects of postoperative radiotherapy to the pelvic, this would be a pro- to postoperative radiotherapy for stage IIIA endometrial carcinoma with isolated adnexa or serosal involvement and should be considered part of the standard treatment. Long-term followup of endometrial carcinoma patients treated with adjuvant pelvic radiotherapy in the PORTEC I study shows us relatively high rates of late morbidity (grade 1) [20]. On the other hand the current study also shows that distant metastasis for stage IIIA endometrial carcinoma with isolated involvement of the adnexa or serosa should be the primary target. Adding effective systemic therapy to surgery and adjuvant radiotherapy might improve the prognosis. Adjuvant systemic treatment for women with high-risk endometrial carcinoma as stage IIIA is a controversial clinical topic that is frequently clouded by strong treatment bias and suboptimal data. Current phase III studies such as the PORTEC III, comparing postoperative pelvic radiotherapy to radiochemotherapy to the pelvis followed by adjuvant chemotherapy, might give some answers in the future. 

Several limitations of this study should be noted. Firstly, it is a retrospective analysis encompassing a 15-year study period; secondly it is a multicentre study, with the possibility of patient selection. Indications for adjuvant external radiotherapy in The Netherlands for FIGO stage IIIA endometrial carcinoma following TAH + BSO were uniform, but the indication for vaginal brachytherapy boost differed between the radiotherapy departments. Despite the relative large number of patients with stage IIIA in this multi-institutional study we were not able to do reliable multivariate analysis in the separate analysis for involvement of adnexa or serosa, making it difficult to tease out the individual contribution of prognostic factors.

## 5. Conclusion

Endometrial carcinoma stage IIIA with isolated involvement of the adnexa or serosa only has a comparable disease-specific survival of 71.8% and 75.4% at 7 years. Locoregional control for patients with adnexal involvement is excellent with only 2.2% recurrences, but worse for patients with serosal involvement at 14.3%. Prognostic factors for DMFS, DFS, and DSS with only adnexal involvement showed to be LVSI, grade of differentiation, and myometrium infiltration. Despite the relative large number of patients with serosal involvement, no prognostic factors could be found. Due to the small number, differences found were not significant.

##  Conflict of Interests

The authors declare that there is no conflict of interests.

## Figures and Tables

**Table 1:** Tumor and treatment characteristics of 67 patients with endometrial carcinoma stage IIIA according to adnexa or serosa involvement.

Characteristics	Adnexa	Serosa	*P* value
	*n* = 46 (%)	*n* = 21 (%)	
Age			
<60 years	21 (45.7)	4 (19.1)	
≥60 years	25 (54.3)	17 (80.9)	.037
Differentiation grade			
Grade 1	23 (50)	7 (33.3)	
Grade 2	11 (23.9)	8 (38.1)	Ns
Grade 3	12 (26.1)	6 (28.6)	
Lymph vascular space involvement			
yes	19 (41.3)	9 (42.9)	
none	27 (58.7)	12 (57.1)	Ns
Myometrium infiltration			
<1/2	21 (43.5)	0 (0)	
>1/2	26 (56.5)	20 (100)	
Cervical involvement			
none	29 (63)	13 (61.9)	
endocervical glands	2 (4.4)	1 (4.8)	Ns
stroma	15 (32.6)	7 (33.3)	
Peritoneal washing			
Positive	7 (15.2)	1 (4.8)	
Negative	15 (32.6)	8 (38.1)	Ns
Unknown	24 (52.2)	12 (57.1)	
Type of radiotherapy			
Pelvic	26 (56.5)	18 (85.7)	
Pelvic + brachytherapy	13 (28.3)	2 (9.5)	Ns
Pelvic + external boost	4 (8.7)	1 (4.8)	
Abdomen/pelvic	1 (2.2)	0	
Abd/pelvic + brachy	2 (4.4)	0	
External dose (no boost)			
30.0 Gy	1 (2.2)	0	
36–45.0 Gy	11 (23.9)	2 (9.5)	Ns
46.0 Gy	34 (73.9)	19 (90.5)	
